# Response to Mahr’s (2026) Response to Wright’s (2025) “Why There Are Exactly Two Sexes”

**DOI:** 10.1007/s10508-026-03452-y

**Published:** 2026-03-26

**Authors:** Colin M. Wright

**Affiliations:** The Manhattan Institute for Policy Research, 52 Vanderbilt Avenue, New York, NY 10017 USA

I welcome Mahr’s (2026) Letter to the Editor critiquing my Commentary (Wright, 2025) on the nature and number of the sexes. Academic disagreement provides an opportunity to clarify points of dispute and potential confusion, and to equip both the scientific community and the broader public with a more accurate understanding of the underlying biology. Because debates about sex are increasingly consequential for public policy, I appreciate the opportunity to respond to Mahr’s specific points. For clarity, I address my disagreements in the order they appear.

Mahr’s first critique is that I allegedly treat the binary “two-and only two” nature of sex as an “a priori” assumption, and that this “presumes a neutral, context-free scientific fact.” Mahr writes that “The very act of defining ‘male’ and ‘female’ by gametes is not a purely descriptive truth but a choice shaped by historical and cultural norms.” While I agree that the *labels* we assign to things are human conventions—we could have used different words than *male* and *female*—the substantive claim at issue is not a label but the observable and recurring natural reproductive phenomenon of anisogamy.

My argument is not “a priori” in the sense of being asserted before observation. It is an a posteriori argument based on the observation that, across anisogamous taxa, individuals participate in reproduction via reproductive systems with the function to produce either small gametes (sperm) or large gametes (ova) (Hilton & Wright, 2023; Minot, 1888). The nomenclature we use for these reproductive classes is a human convention, but the underlying biology is not. Here’s an analogy: Humans named “water” long before we knew its composition, and only later discovered through experiment that it is a molecule composed of two hydrogen atoms and one oxygen atom (H_2_O) (Edelstein, 1948; Eisenberg & Kauzmann, 1969). We could have chosen a different name for the molecule, but its structure was what it was before we discovered it and would remain the same regardless of what we called it. Likewise, the two reproductive strategies based on producing either small and large gametes predate any human categorization by over a billion years (Butterfield, 2000).

Mahr then rejects my claim that the gamete-centered definition is objective or universal, calling such claims a “god trick” and an “illusion of view-from-nowhere,” and appealing to feminist epistemology to argue that scientific categories are historically and socially situated. In the broad sense, science is indeed a human activity embedded in history. But it does not follow that biology cannot therefore describe objective, mind-independent features of the world. Many scientific claims are objective in the relevant sense: The Sun sits at the center of our solar system (Copernicus, 1543); water is composed of hydrogen and oxygen in a 2:1 ratio (von Humboldt & Gay-Lussac, 1805). Humans had to discover these facts and represent them through language and models, but their truth is not dependent on culture. Likewise, male and female as reproductive classes based on gamete size are an observable, general phenomenon that makes these categories coherent, comparable across taxa, and explanatorily useful (Hilton & Wright, 2023). The question is not whether or how scientists are “socially situated,” but whether the proposed classification tracks a real and recurrent functional distinction in nature. In anisogamous species, it does.

Mahr next argues that a gamete-based definition “ignores the many other biological features that vary (chromosomes, hormones, brain structures, etc.),” and claims that the “biological elements commonly linked to ‘sex’ (including cells, tissues, molecules, structures, and pathways) do not neatly divide into two distinct categories.” From this, Mahr concludes that defining sex “strictly by gamete size is a reduction that omits these complexities.” This objection is addressed directly in my original Commentary’s discussion of “polythetic” accounts of sex (Wright, 2025). There, I described the polythetic view as one in which sex is treated as “an aggregate of traits—chromosomes, gonads, gametes, hormones, neuroanatomy, secondary sex characteristics, and other sexually dimorphic traits—and individuals are assigned degrees of maleness or femaleness according to how their overall profile aligns with what is considered male-typical or female-typical.”

The difficulty with this view is not that trait variation is uninteresting, but that polythetic models conflate determinants and downstream correlates of sex with sex itself. As I wrote, “Traits are labeled ‘male-typical’ or ‘female-typical’ because they correlate with males and females already identified independently—ultimately by reference to gametes.” In other words, the polythetic view collapses in on itself because it depends on the very classificatory foundation—male and female as functional reproductive classes rooted in gametes—it seeks to replace.

The gamete-based definition does not deny biological variation; it organizes it. Chromosomes, hormones, genital development, and many dimorphic traits vary—sometimes substantially—within each sex, and development can produce atypical outcomes. But variation in *correlates* of sex is not the same thing as variation in sex itself. In anisogamous organisms, the universal distinction that makes “male” and “female” biologically comparable across taxa is reproductive function with respect to sperm versus ova (Bogardus, 2025). This is also why it is a mistake to treat any of these *correlates*, whether individually or collectively, as constitutive of sex.

For instance, there is nothing inherently “male” about a Y chromosome as such. In mammals, the Y chromosome happens to play a central role in triggering male development (Wilhelm et al., 2007); in birds, the sex chromosomes differ (ZW systems) (Ellegren, 2000); and many reptiles lack sex chromosomes entirely (Janzen & Paukstis, 1991). Yet despite the diversity of sex determination mechanisms (Bachtrog et al., 2014), the sex classes across taxa remain intelligible and comparable because they remain anchored in gamete size. This is also why sex change in sequential hermaphrodites is intelligible: We identify the transition precisely by a shift in which type of gamete the organism’s reproductive system has the function to produce (Avise & Mank, 2009). Without gametes as the universal defining trait, “male” and “female” lose all cross-taxon coherence and become nothing more than meaningless labels attached to shifting bundles of correlates.

Mahr then takes issue with my claim that definitional clarity has “scientific and societal benefits,” arguing that “rigid essentialism about sex often undergirds exclusionary norms,” including when “policy-makers or courts cite ‘science’ to restrict transgender rights.” This concern highlights an important distinction. One issue is descriptive: What an individual’s sex *is* as a biological classification in anisogamous species. Another is normative: What rights or accommodations a society ought to adopt for males and females. My claims about the number of sexes and how they are defined are not grounded in a preference for any policy outcome, but in what makes the categories biologically coherent and explanatorily useful. People are free to advocate for many different policies regarding sport, bathrooms, or even social recognition. But while biology can and should inform policy, policy preferences should never be used to dictate biology.

Finally, Mahr writes that the binary view “risks invalidating the lived experiences of intersex and gender-diverse individuals,” references “non-binary genders,” and invokes the claim that “a large body of research shows that about 2% of people have intersex traits that challenge a simple XX/XY schema.” My Commentary was intentionally about the biology of sex, not gender identity, roles, or expression. Those are separate topics that are irrelevant to how many sexes there are and how they are defined in evolutionary and reproductive terms.

With respect to the claim that “about 2% of people have intersex traits that challenge a simple XX/XY schema,” it is both misleading and, as presented, poorly supported by the citations provided (Fausto-Sterling, 1993, 2025). The widely repeated “2%” figure is arrived at by adopting an overly expansive definition of “intersex” as deviation from a “Platonic ideal of physical dimorphism at the chromosomal, genital, gonadal, or hormonal levels” (Blackless et al., 2000). Because conforming to a “Platonic ideal” is an impossibly high standard, this approach dramatically inflates prevalence by counting many non-ambiguous conditions as “intersex.” In response, Sax (2002) argued that if “intersex” is to retain a clinically meaningful definition, it should be “restricted to those conditions in which chromosomal sex is inconsistent with phenotypic sex, or in which the phenotype is not classifiable as either male or female.” Under this narrower, realistic, and clinically useful definition, the prevalence estimate drops two orders of magnitude to approximately 0.018%. However, the existence and prevalence of DSDs or “intersex” conditions are irrelevant to the question of how many sexes there are. While developmental variation may produce sexually ambiguous phenotypes in extremely rare cases, they do not constitute additional or intermediate sexes because they do not result in reproductive anatomy with the function of producing novel gamete types beyond sperm and ova.

In sum, Mahr’s critique is mainly about the sociology of categories and the policy implications of scientific debates. Those are fair topics to discuss, but they do not change the biological basis for how the sexes are defined. The sex binary is not an “a priori” assumption, but an empirical observation that there exist only two distinct gamete types in anisogamous species. Treating sex as a polythetic bundle of correlates confuses the determinants and downstream effects of sex with sex itself, and ultimately relies on the gamete foundation it seeks to undermine.

The renowned geneticist, Dobzhansky (1973), wrote that “Nothing in biology makes sense except in the light of evolution,” highlighting evolution as the unifying framework that makes life’s diversity, complexity, and interconnectedness comprehensible. I contend that a parallel statement applies similarly to reproductive biology: Nothing in the biology of the sexes makes sense except in the light of gametes.
